# Maximizing the value of patient and public involvement in the digital health co-design process: A qualitative descriptive study with design leaders and patient-public partners

**DOI:** 10.1371/journal.pdig.0000213

**Published:** 2023-10-25

**Authors:** Paula Voorheis, Jeremy Petch, Quynh Pham, Kerry Kuluski

**Affiliations:** 1 Institute for Health Policy, Management, and Evaluation, University of Toronto, Toronto, Canada; 2 Centre for Data Science and Digital Health, Hamilton Health Sciences, Hamilton, Canada; 3 Division of Cardiology, Faculty of Health Sciences, McMaster University, Hamilton, Canada; 4 Population Health Research Institute, Hamilton Health Sciences, Hamilton, Canada; 5 Centre for Digital Therapeutics, Techna Institute, University Health Network, Toronto, Canada; 6 Telfer School of Management, University of Ottawa, Ottawa, Canada; 7 Institute for Better Health, Trillium Health Partners, Mississauga, Canada; Universität Wien: Universitat Wien, AUSTRIA

## Abstract

Digital health interventions have enormous potential to support patients and the public in achieving their health goals. Nonetheless, many digital health interventions are failing to effectively engage patients and the public. One solution that has been proposed is to directly involve patients and the public in the design process of these digital health interventions. Although there is consensus that involving patients and the public in collaborative design is valuable, design teams have little guidance on how to maximize the value of their collaborative design work. The main objective of this study was to understand how the value of patient and public involvement in digital health design can be maximized, from the perspective of design leaders and patient-public partners. Using a qualitative descriptive methodology, we conducted semi-structured interviews with 19 design leaders and 9 patient-public partners. Interviewees agreed that involving patients and the public was valuable, however, they questioned if current collaborative methods were optimized to ensure maximal value. Interviewees suggested that patient and public collaborative design can add value through four different mechanisms: (1) by allowing the design process to be an empowering intervention itself, (2) by ensuring that the digital health intervention will be effectively engaging for users, (3) by ensuring that the digital health intervention will be seamlessly implemented in practice, and (4) by allowing patient-public collaborations extend beyond the initial product design. Overall, interviewees emphasized that although collaborative design has historically focused on improving the digital health product itself, patients and the public have crucial insights on implementation planning as well as how collaborative design can be used as its own empowering intervention. The results of this paper provide clarity about the ways that patient and public collaborative design can be made more valuable. Digital health design teams can use these results to be more intentional about their collaborative design approaches.

## Author summary

Digital health intervention development could benefit from involving patients and the public in the design process. Until now, digital health design teams have received little guidance on how they can involve patient-public partners in the most valuable way. In this paper, we sought to understand how to maximize the value of collaborative design with patients and the public by interviewing digital health design leaders and patient-public partners. Interviewees suggested that there are four different ways collaborative design adds value: (1) by allowing the design process to be an empowering intervention itself, (2) by ensuring that the digital health intervention will be effectively engaging for users, (3) by ensuring that the digital health intervention will be seamlessly implemented, and (4) by allowing patient-public collaborations extend beyond the initial product design. Until now, patients and public involvement has been focused on improving the design of the digital health product itself. Although this is important, interviewees emphasized that patients and the public have crucial insights on implementation planning as well as how collaborative design can be used as its own empowering intervention. The results of this study can help design teams improve their collaborative design in the future.

## Introduction

### Background

Many experts in digital health feel that we are at a turning point in digital health design [1]. Although COVID-19 has advanced digital health innovation quicker than previously imagined [2], we are only beginning to realize the potential of what digital health can offer [3]. Although digital health has enormous potential to leverage expertise from diverse health sectors and increase the reach of health services, digital health design is fragmented and seems to be missing the mark in terms of effectively engaging patients and the public [3–5]. With > 90,000 health applications developed in 2020 alone, we appear to be in the midst of a design explosion with little directionality or coordination [6]. Although the intent of digital health is to improve patient and public health, we have taken little time to pause and question if were doing things right. Are we ensuring that the digital health solutions we are designing are adding value for patients and the public?

One solution that has been proposed to improve digital health design is to directly involve patients and the public in the digital health design process [7–11]. User-centred design [11], human-centred design [12,13], collaborative-design [8,14], participatory design [10,15,16], and other related design methods all aim to put patient and public end-users at the forefront of design. As of now, there seems to be a consensus that involving patients and the public in the digital health design process “adds value” [11,17]. For instance, the digital health design field conceptualizes patient and public involvement as a way to improve the user-centredness of digital health solutions, thus increasing user engagement and intervention effectiveness [11,17]. Considerable work has been done to clarify the *principles* of patient and public involvement (e.g., fostering respect) [18,19] as well as the *methods* that can be used to involve patients and the public (e.g., brainstorming workshops) [8,9]. Although these advancements have been useful for advancing involvement practices, questions remain around how digital health design teams can *maximize the value* of patient and public involvement [20,21]. Specifically, there seems to be a “black box” around the specific ways in which collaborative design with patients and the public can be made more valuable.

In a previous scoping review, our authorship team found that digital health designers often struggled to include patient and public partners throughout their design process, and if they did, there was concern whether involvement was a “check-box item” with little value-add for everyone involved [22]. Recent work by Noorbergen et al. has provided practical guidance to help digital health design teams operationalize collaborative design approaches, offering strategies to help teams overcome some common challenges of patient and public involvement [14]. Their work proposes specific collaborative design recommendations, which can be mapped onto typical design phases. Although Noorbergen et al. have provided a practical step forward, their work does not clarify *the value* that should be created through these collaborative design approaches. Their work also is based on the perspectives of design leaders only, rather than design leaders and patient-public partners together. To our knowledge, the digital health literature has yet to clarify the specific ways that the value of collaborative design with patients and the public can be maximized, from the perspective of design leaders and patient-public partners.

### Research question and objectives

This paper aims to answer the following overarching research question: How can the value of patient and public involvement in digital health design be maximized, from the perspective of digital health design leaders and patient-public partners?

Our research team decided to focus our research question on “maximizing value” for several reasons. First, the results of our previous scoping review suggested that many design teams are still unsure about how to involve patients and the public in the most valuable way [22]. Second, members of our research team have experienced situations where they wished they could have had more guidance on how to maximize value in their own collaborative design efforts. Overall, our research team felt that clarifying how to maximize the value of patient and public involvement would be an important advancement to the literature. Until now, digital health design teams have received little guidance on how they can involve patient-public partners in a value-maximizing way.

## Methods

### Methodological orientation

This research is founded on naturalistic inquiry, which involves using rich descriptions of participant perspectives while maintaining an objective to advance practice [23,24]. The ontological position of naturalistic research is fundamentally relativist; recognizing that reality is subjective and varies across participants [24]. Our research team embraced constructivist and pragmatist epistemological perspectives, which are aligned in their recognition that knowledge is socially constructed by participants and researchers [25,26]. To correspond with our epistemological perspectives, the tenants of naturalistic inquiry, and the overarching research question, our research team selected a qualitative description methodology, which involves obtaining minimally theorized answers to research questions in applied settings [23,24]. Qualitative description is particularly appropriate in this study given our descriptive research aim and pragmatic desires to improve digital health design methods. The COREQ checklist [27] and guidance for publishing qualitative research in health informatics [28] helped us ensure rigor in our reporting. This paper refers to “patient and public involvement” interchangeably with “patient and public engagement” and “patient and public collaborative design (i.e., co-design)”, as these terms were used by interviewees.

### Research team

The lead researcher is a PhD candidate at the University of Toronto (PV; she/her). Her work focuses on improving the design methods of digital health applications, and she has a background in behavioural science and qualitative methods. She conducted all the qualitative interviews and led the data analysis. She had no prior relationships with the interviewees except for two patient partners, who she had met in a prior collaborative design workshop. The remainder of the research team is comprised of three professors from the University of Toronto with expertise in patient and public engagement (KK; she/her), digital health (QP; she/her) and artificial intelligence (JP; he/him). All members of the research team believe that involving patient-public partners in digital health design is valuable.

### Participants

We aimed to recruit two types of participants: “digital health design leaders” and “patient-public partners”. Design leaders had to (a) have designed a digital health intervention that aimed to facilitate health-related behaviours in patients or the public, and (b) have engaged patients or public end-users in the digital health design process. Patient-public partners had to have been involved in the design process of a digital health intervention. This involvement could have been throughout the entire design process or just during one stage. It should be noted that many design leaders had experiences being a patient or caregiver (e.g., they were living with a health condition or were caring for a family member with a health condition), and many patient-public partners had experience with intervention design (e.g., they were previous healthcare or information technology managers). This blurring of participant groups was not unexpected, as individuals are complex and are likely hold multiple roles in their life. When we interviewed participants from these different recruitment groups, we aimed to ask them questions pertaining to their role as either a design leader or patient-public partner. We aimed to recruit participants who were involved in designing digital health interventions for diverse health issues, across a range of geographic locations, and in both academic and industry settings. We used purposeful and snowball sampling to guide our participant recruitment. The goal of purposive sampling in qualitative description is to obtain participants who will provide information-rich data in reference to the research question [23]. Purposive sampling was guided by the results of our previous scoping review [22] and by the expert knowledge of our research team. Snowball sampling allowed us to ask participants if they knew design leaders or patient-public partners who would be appropriate for our study. Individuals who were thought to meet our inclusion criteria were contacted by email. The final interviewee sample size was determined by “conceptual depth”, where we continued iterative data collection and analysis until we felt we had sufficient richness in information related to the research question [29]. A full description of the interviewees can be found in **S1 Appendix.** Pre-interview demographic data (e.g., gender and race-based data) were not collected from interviewees. Limitations associated with our lack of pre-interview data collection are outlined in the discussion section. Reasons for non-participation included non-response and denial due to previous publication on the topic.

### Data collection

Single, semi-structured interviews with design leaders and patient-public partners were conducted over Zoom software using an interview guide with open-ended questions [23]. Questions were purposely left open-ended to allow for the interviewer (PV) to follow up on important threads in the discussion. The interview guide was iteratively drafted over the course of several meetings with our research team. The interview guide was pilot tested with members of a digital health design team currently partnering with patients and the public. The questions in the interview guide are summarized in Table 1. Discussion on the topics in Table 1 lasted approximately 30 minutes. Interviews were audio recorded and transcribed with the aid of Otter.ai software. The interviewer (PV) also took notes during the interviews, which were included alongside the transcripts.

**Table 1:** Semi-Structured interview guide sample questions.

Interviewee	Open-Ended Question Prompts
Design Leaders	• Why was it important for you to involve patient-public partners? ○ What was the value-add? • How did you engage patient-public partners in design? ○ How was value-added? • What do you think was done well and what could have been done better? ○ How was value maximized and how could value be increased further? • What do you think is needed moving forward to make collaborative design more valuable? ○ What are the best ways to ensure value is maximized?
Patient-Public Partners	• Why were you brought onto the digital health design process? ○ What was the value-add? • How were you engaged in the design process? ○ How was value added? • What do you think was done well and what could have been done better? ○ How was value maximized and how could value be increased further? • What do you think is needed moving forward to make collaborative design more valuable? ○ What are the best ways to ensure value is maximized?

### Data analysis

Our research team followed an inductive thematic analysis approach, which was appropriate given our qualitative descriptive methodology and aim to describe participants’ own perceptions [30]. Specifically, we followed the reflexive thematic analysis described by Braun and Clarke [30–32]. Our reflexive thematic analysis process involved (a) gaining familiarity with the data, (b) generating initial codes, (c) generating initial themes, (d) reviewing and developing themes, (e) refining, defining, and naming themes, and (f) producing the report [30]. To gain familiarity with the data, the lead researcher, PV, reread the transcripts and interview notes. To generate initial codes, PV worked through the dataset, focusing on making succinct labels relevant to the research question. During this process, KK and PV coded three interview transcripts together (two from design leaders and one from a patient-public partner) and reflexively discussed their interpretations of the data. After further discussion about aggregating individual codes into wider meanings across the dataset, PV moved into theme generation. NVivo 11 was used to collapse multiple codes that shared a similar underlying concept into single codes with a richer meaning. PV subsequently began creating a thematic map to tell a wider analytic story with the data about how the value of patient and public involvement could be maximized during digital health design. PV presented candidate themes and sub-themes to the research team, where overlapping and tangential themes related to the research aim were refined. Once the themes were agreed upon, the research team defined and named these themes, ensuring the language was precise and coherent. A qualitative report was subsequently produced, and participants were recontacted to review a report abstract. Trustworthiness in the data analysis process was ensured through several approaches, including reflexive notetaking, peer-to-peer debriefing, thematic diagraming, recontacting interviewees, and reporting quotations alongside thematic descriptions. In addition, the lead researcher PV engaged in activities to enhance her reflexivity, including listening to podcasts about patient and public engagement and attending conference presentations focused on collaborative design methods.

### Ethical approval

This study was approved by University of Toronto Research Ethics Board (REB #42515). To respect that some participants wanted to be acknowledged for their contributions to this paper, we obtained ethical approval that allowed interviewees to decide whether they wanted their name to be recognized or remain anonymous. Specifically, interviewees could have (a) their name attached to direct quotes, (b) their name recognized for their general contributions, or (c) their name kept anonymous.

## Results

### Participants

Rather than being described through general descriptors, most interviewees requested to be directly recognized for their contributions to the paper. A detailed description of the interviewees is provided in **S1 Appendix**. Overall, 28 individuals were interviewed for this research, including 19 design leaders and 9 patient-public partners. Interviewees were in multiple locations, including 16 from North America, 8 from Europe, and 4 from Oceania. Interviewees were involved in designing several different types of digital health interventions, such as interventions aimed to support individuals with cardiovascular disease, diabetes, cancer, depression, joint pain, rheumatoid arthritis, multiple sclerosis, smoking addiction, and multiple chronic conditions, and hospital-to-home transitions.

### Themes

**Fig 1:**
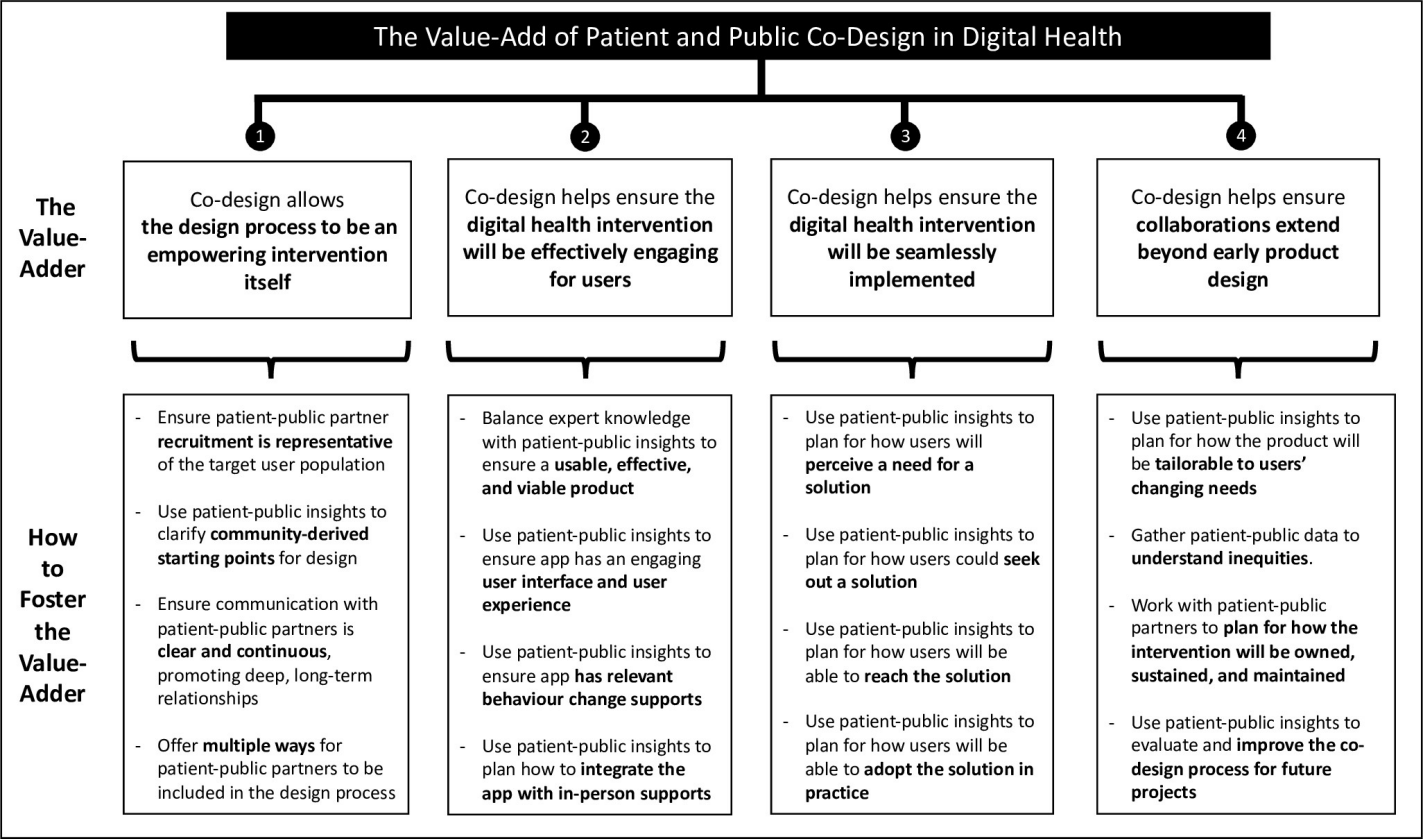
How Patient and Public Co-Design Adds Value to Digital Health Design.

Design leaders and patient-public partners largely agreed that involving patients and the public in the design process of digital health was “valuable”. However, interviewees questioned if current patient and public collaborative design methods were optimized to ensure value was maximized. Interviewees suggested that patient and public collaborative design should be viewed as adding value through four different mechanisms; (1) co-design adds value by allowing the design process to be an empowering intervention itself, (2) co-design adds value by ensuring that the digital health intervention will be effectively engaging for users, (3) co-design adds value by ensuring that the digital health intervention will be seamlessly implemented in practice, and (4) co-design adds value by creating a foundation for patient-public collaborations to extend beyond the initial product design. Fig 1 summarizes these value-adders with detail on how this value can be cultivated.

#### 1. Co-Design: Allowing the design process to be an empowering intervention in itself

Both design leaders and patient-public partners recognized that the design process could be seen as an intervention itself, giving patients and the public a platform to share their stories and feel hopeful about their health journey.

*They have the patient or caregiver partner come up and tell their story to kick off a meeting. [Other stakeholders] realize, “oh, I never heard that perspective before”. I find it particularly rewarding to just see the difference I make in being able to tell my story and people understanding.–Patient-Public Partner*

Interviewees shared several ways to facilitate an empowering design process. First, most interviewees felt it was essential to recruit patient-public partners who were representative of the target population, which often meant venturing outside of one’s organization. Patient-public partners often felt they were overly relied on and often not the best choice for designing a solution that would truly fit end-user needs.

*The patient advisors who were brought on to the project were all very able-bodied and had no reason to use the app. […] No offense to you to all you researchers, but you’re in an office. You have to go out there and find your candidates.–Patient-Public Partner*

*I know they were looking for people to participate in the co-design of the [digital health project], but I declined to do that, because I’ve been involved with so many things already. I thought it would be good to have a fresh perspective.–Patient-Public Partner*

Interviewees also commented that design leaders too often start the design process with a deficit mindset, assuming that the target population is doing things wrong and needs new supports. Interviewees suggested that it is important to work with patients and the public to clarify more community-derived starting points for interventions.

*In my experience*, *designers [from behavioral science] are often coming with perspective of*, *“we want to solve this problem” and “people are doing the wrong things”*. *I’ve never liked that approach*. *The way I like to approach things is*, *“what are people’s individual goals or familial goals or community goals*?*” I often work with people as individuals*, *but I also recognize that people exist within families and communities that may influence their own individual choices*. *[…] How do we help build tools that can support this*, *rather than the thing they’re doing wrong*?–*Holly Witteman*, *Design Leader*

Interviewees also reflected that for co-design to feel empowering, patient-public partners need to feel a part of the team. There must be iterative, ongoing communication throughout the entire design process, especially during project down-times and personnel changes.

*[Jane Doe] was the project manager*. *She was just fantastic with us*. *But eventually [Jane Doe] left and went off to work in another area*. *And so then we had an interim project manager*. *[…] She was not very good*. *[…] She will always keep the clinicians in the loop*. *Sometimes we have to kind of keep reminding her “Hello*, *we are here”*. *So it’s very important to make sure that patients aren’t seen as these little nice-to-haves*–*Patient-Public Partner*

Overall, interviewees agreed that design teams need to prioritize how co-design methods can be tailored to fit patient-public involvement needs, rather than just how co-design methods can be used to elicit the most information for the digital health app.

*In recruitment*, *you should be thinking about all the different patients*, *and making sure that there’s more than one way for people to be engaged*. *[…] I wasn’t always available to attend the formal meetings*, *so they really accommodated me*. *I was able to look at minutes and send my thoughts through email or meet with them later on*.–*Patient-Public Partner*

#### 2. Co-Design: Ensuring the digital health intervention will be effectively engaging for users

Although patient-public partners wanted to feel prioritized in the design process, they also wanted the resulting digital health intervention to be effective. Patients and the public wanted their inputs to make a difference, and often recognized that they couldn’t be the experts in many design situations. Design leaders agreed and suggested the following:

*One of the things that I see coming out of the patient community is that patients really need to be put in the driver’s seat*. *[…] I agree*, *however*, *in my experience*, *I also think there’s a role for the expert designer to take their learning from the patient and transform that into something that’s a viable product*.–*Design Leader*

Interviewees discussed several ways patient-public inputs could be integrated to help create a more effectively engaging digital health design, which included improving the intervention’s (a) user interface and user experience, (b) health behavior change supports, and (c) integration with existing in-person supports. Regarding the user interface and user experience, patient-public insights are essential to provide designers with strategic direction on how the product can be made simple, relatable, and easy to use.

*In the development of [digital health] apps*, *I think it’s about keeping them simple*. *Giving people the access to the right information*, *for them*, *quickly*, *without making it onerous*, *or overwhelming people*. *I’m the person that wants to dive in*, *I’ve got an analytic nature*. *That’s me*, *but not everyone wants that… We have to work together to find the “sweet spot” that speaks to many people as we can*.–*Patient-Public Partner*

Regarding the design of relevant health behavior supports, design leaders generally found it helpful to use behavioral science frameworks to make sense of patients’ behavioral barriers and facilitators. Nonetheless, design leaders reflected that these frameworks might be limiting the ideation of appropriate solutions, and that community-derived frameworks of behavior change may need to be considered.

*I very naively took the exact behavioral science models that I used before*, *and was like*, *“well*, *we could just use them here”*. *When we applied them*, *we quickly realized that none of the solutions using that behavioral science framework resulted in anything fruitful*, *or anything that could actually be implemented*, *or anything that people felt would work*.–*Design Leader*

Regarding integration with existing in-person supports, several patient-public partners commented that they felt the value of their inputs extended beyond the digital product itself. Interviewees wanted to use their insights to help design how the product would be mixed with other existing healthcare interventions, especially those delivered in person.

*Over the past three years*, *my trainer has been on Zoom*. *But there is still a person that that I know*, *who is waiting for me at a certain time*, *and we connect by Zoom*. *And that’s what the technology provides and it’s great*. *But it’s a real person there*. *That’s the intervention*. *If I went on an app that just showed me an exercise*, *I know that I wouldn’t do it*. *The way to go is to marry them in some way*.—*Patient-Public Partner*

Overall, design leaders and patient-public partners agreed that effective design necessitates finding a balance between several different forms of evidence to create a well-rounded digital health intervention. Interviewees agreed it is important to be transparent with patient partners about how their voices will be balanced with other inputs and knowledge.

*I think the main thing is how to find the perfect balance between all of the inputs from the start of a project. Community involvement in the co-design process is incredibly powerful and really important. But you can’t let it completely outweigh what you know works and what you’ve found doesn’t work. […] There’s got to be more balance between all of these bits–Design Leader*

#### 3. Co-Design: Ensuring the digital health intervention will be seamlessly implemented

Although involving patients in the co-design of the digital health intervention itself was viewed as important by most interviewees, many patient partners also wanted to be involved in co-designing how the solution would be promoted and implemented in practice.

*But when [the app] got up and running*, *I think there was too much effort put into the program as opposed to the recruitment and getting the uptake by the clinicians*.–*Patient-Public Partner*

Many interviewees commented that our current approaches to co-design focus too much on the product design, rather than on how the digital health intervention will be supported and disseminated in context.

*I think it really comes back to thinking about how it’s actually going to roll it out at scale*. *You want to test the whole process of how do you get people into it*? *How do they sign up*? *How do you find them in the first place*? *And then how do you give them a program that they’re going to stick with over the period that you want them to stick with it*?–*Design Leader*

Interviewees proposed several approaches for how patients and the public could be involved in co-designing a digital health implementation plan. First, interviewees suggested that design teams need to discuss with patient-public partners how the target population will even perceive there to be a problem in the first place.

*In a place like remote Ethiopia or rural Kenya*, *communities are constantly moving around*. *They’re not on their cell phones checking their health*, *if they’re even concerned about their health at all*. *I think that the basis that you’re starting on is very different*. *[…] It moves beyond*, *“Oh*, *we need to make this small*, *subtle tweak in our app to increase confidence in completing a medical exam”*. *It goes more into*, *“We want to get people to do medical things in the first place*. *[…] What supports them to do exams in their regular everyday lives*?*”*–*Design Leader*

Interviewees also agreed that patients and the public need to be involved in helping plan for how the target population would eventually seek a solution, leveraging their own local communities and internal networks.

*It’s the marketing and the “what’s in it for me*?*” If you think about prostate cancer*, *the age group is getting older*, *and they just can’t be bothered anymore*. *They lose interest*. *And so how is it marketed to them*? *I say it succeeds when it’s about “it’s not just for us*, *but it’s other men out there”*.–*Patient-Public Partner*

Even if patients and the public decided that they would be interested in a digital health solution, interviewees reflected that it might be complicated for the target population to actually reach that solution.

*There’s still probably going to be access issues*, *right*? *Because does everyone have the ability to get on the app*? *Does everybody have a cell phone*? *I still think there’s some socio-economic groups that would have difficulty accessing an app*.–*Patient-Public Partner*

Patient and the public can help design teams understand that the target population may have individuals with multiple health concerns and access issues. Even if the individual app is patient-centered, the combination of four or five patient-centered apps that don’t integrate with each other may lead to failure.

*Unless we change something, we are going to have a different app for everything and all of them with different logins. They won’t speak to each other, and that will be very detrimental for the patient. Especially when we’re speaking mostly about people with chronic conditions that tend to be older and tend to have literacy issues. And maybe they also have cognitive impairment to some level. If we don’t simplify this, we will actually be increasing health inequity, rather than decreasing it.–Design Leader*

Overall, interviewees reflected that co-designing the implementation strategy should happen at the start of the digital health design process, and should be considered just as important as co-designing the actual digital health solution.

*The implementation focus from the beginning has very clearly been about getting all the right stakeholders on board from the start… At the end*, *to suddenly say*, *“I’ve got this fantastic thing and it works*. *Now you should fund it and own it and put it out there”*, *they’re going to be like “Oh*, *well*, *were not quite sure”*.–*Design Leader*

#### 4. Co-Design: Allowing patient-public collaborations to extend beyond early product design

Given rapid developments in digital health technology, many design leaders and patient-public partners expressed that the co-design process should never actually end. Interviewees expressed that digital health interventions should be designed in a way that allows patients to tailor the product to align with their changing needs over time.

*It has to be that the app doesn’t do everything all at once*. *You don’t want to start with an app that tells you what to do with your diet*, *what to do with your exercise*, *what to do for cognitive stimulation*, *what to do that’s social*, *and also deals with your smoking and your drinking*. *[…] What’s the one thing you want to start with*?—*Patient-Public Partner*

Interviewees commented that by allowing for apps to be tailorable, we can start thinking of co-design as a way to allow for ongoing patient inputs to be acknowledged, especially inputs that may reduce inequities in care.

*And we know that some percentage of that population is experiencing poor social determinants of health*, *so we want to be able to offer them solutions to the barriers related to that*. *And so what we’re doing is we’re trying to build a library of content that’s inclusive of all of these sorts of barriers and then use our AI to actually identify which individuals are in need of that content*.–*Design Leader*

The implication of the design process never ending means that someone must be responsible for its ownership, adaptation, and sustainability. Even if an intervention is patient-centered during its initial launch, an inability to update itself means that it may quickly become non-patient centered.

*One of the biggest challenges that we’ve had is once we get the development out*, *we’re coming onto ongoing software updates*. *One of the things we’ve learned is that you need to monitor participants use on the app in real time*, *and identify when user like falls off*.–*Design Leader*

A long-term commitment to upkeep a digital health intervention means that it must be perceived as worthy of investment. By involving patients and the public in co-design, patients may feel a greater sense of ownership and may not necessarily care if it is or is not leading to evidence-based health changes.

*At the end of the study*, *we said*, *“well as the academics in this group*, *the app didn’t work*, *so we can’t go around promoting it now”*. *However*, *the community was like*, *“well*, *we don’t care*. *We love it*. *We want to use it and we want to implement it*. *We realize it’s not going to work for everyone*, *but for the people who use it*, *they will love it and it will work for them*. *You gave us ownership*, *so we want to own it*.*”*–*Design Leader*

Overall, given the pace of change in digital health, we need to think about future-proofing our co-design methods so that the interventions we create will not fall out of favor. Creating adaptive digital health solutions will require co-design processes that are ongoing and allow for continuous improvement. Patient-public partners can help us understand what data we should be using to create the most appropriate solutions over time.

*You have to be able to pivot*, *constantly*. *Technology will definitely keep changing*. *We don’t know what it will be*, *but we know it’d be different*. *[…] What’s really important is the process more than the result […] I think your research needs to spend more time on perfecting the design process and not the platform*.–*Patient-Public Partner*

## Discussion

### Primary findings

This paper presents a new conceptualization of how the value of patient and public involvement in digital health design can be maximized. Previous work has provided insight on what meaningful patient and public involvement entails, including ensuring patient-public partners feel respected, are included in team interactions, and are given clear roles and responsibilities [19,33,34]. Although these clarifications help guide how patient-public partners should be treated, they lack connectivity with how this respectful treatment can be used to enable patient-public partners to make a valuable impact. The results of this paper results suggest that it was not enough for patient-public partners to simply feel respected in the collaborative design process itself. Patient-public partners wanted their contributions to lead to change in the digital health intervention. Our results suggest that patient-public partners can offer important insights on the digital health product’s user interface design, user experience design, behaviour change design, and its integration with existing in-person supports. Patients and the public also have important insights on the digital health product’s implementation, including how other patients would perceive there to be a problem, would be able to seek the solution, would need support to reach the solution, and would access the solution in its full capacity. Several patient-public partner interviewees stated that they wished they could have been more involved in the digital health product’s implementation planning, as they had distinctive knowledge about how their own communities function. The results of this paper suggest that current digital health co-design efforts may be underappreciating the role that patient-public partners play in implementation design. Similar findings were recently noted in a paper by Papoutsi et al., where they described “if co-design focuses narrowly on the technology, opportunities will be missed to coevolve technologies alongside clinical practices and organizational routines” [35]. The results of this paper, alongside Papoutsi et al.’s work, suggest that the current literature may not be clear enough about the different ways that patient and public co-design value can be maximized.

Another primary finding of this paper is that co-design should be viewed as important intervention in itself, in addition to its value for digital health intervention design and implementation. The results of this paper suggest that co-design might be eliciting an intervention-like effect by being a source of empowerment, increasing patients’ ability to engage in health-related behaviours. Patient-public partner interviewees stated that being involved in co-design allowed them to share, listen, connect, and feel in more control over their health. Consequently, it might be advantageous for digital health design teams to not only use co-design as a way create a more effective digital health intervention, but also use co-design as a way to foster an environment of empowerment among a target population who may deeply benefit. Although this profounder impact of co-design is encouraging, there is the obvious issue of scalability; design teams can only meaningfully involve a small subset of target population in initial co-design activities. The issue of co-design scalability leads into another primary finding of this paper which is that interviewees felt that the co-design process should never actually end. Interviewees suggested that we should make co-design an ongoing capability throughout the product’s lifespan where all users can control and tailor the product’s content and features over time. Our research team found this result interesting because of its relationship with how other successful digital applications function (e.g., non-health applications such as Instagram, TikTok, Twitter, Facebook, and health-specific applications such as Calm, Headspace, Strava, and Noom). All these applications have an element of enduring co-design, where users are not just passive receivers of content, but can constantly adapt the platforms to meet their own needs. In these applications, all users can create a digital experience that feels like their own. Thus, it is worth considering whether digital health teams should further prioritize designing solutions that are more adaptable for users, making them all feel like they are, inherently, co-designers. However, to appropriately decide on which content and features should be tailorable (and how they should be tailorable), early co-design efforts are still needed to ensure these tailoring mechanisms are appropriate for the target population. The appropriateness of tailoring capabilities (e.g., in language, messaging, imagery, etc.) relates another primary finding of this paper, which is the importance of recruiting representative users into the co-design process. It is worth considering that if design teams do not recruit representative users into the co-design process, not only may they fail to create an appropriately tailored digital health intervention, but they also may fail in allowing the co-design to be an impactful intervention in itself for a deserving target population. It is widely recognized that despite equity-deserving populations being the most affected by health behavioural issues, these populations also the least likely to be involved in co-design efforts [36]. By not dedicating considerable time and effort into patient and public partner recruitment, design teams may be missing out on creating a co-design environment that can help increase the confidence, engagement, and autonomy of equity-deserving populations.

Overall, the results presented in this paper offer a helpful starting point for digital health designers to think more critically about how they can involve patients and public in a way that maximizes value. Hopefully the specificity provided in this work will allow design teams to be more intentional about their co-design practices in the future.

### Recommendations

**Fig 2:**
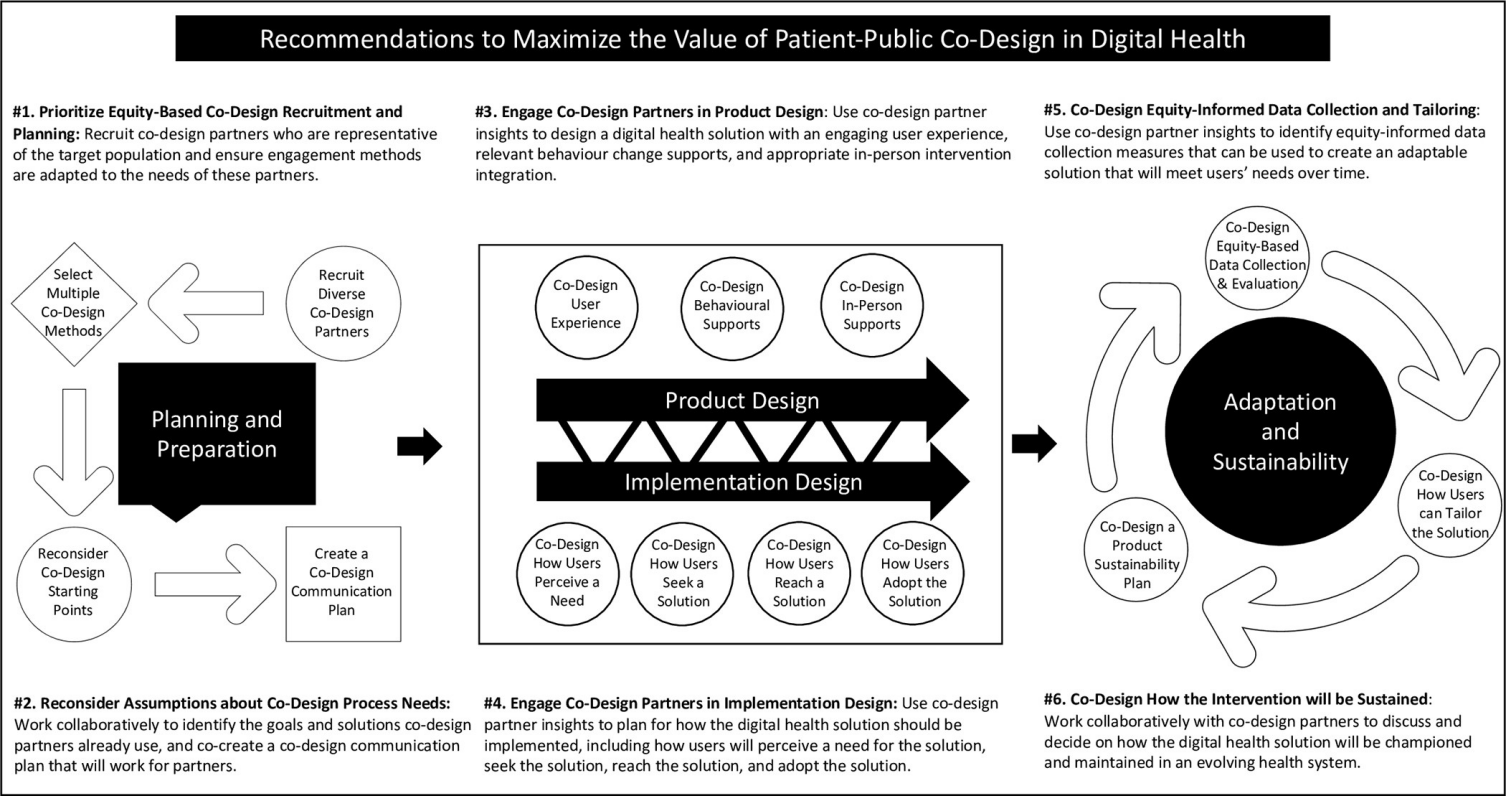
Recommendations for How to Maximize the Value of Patient-Public Co-Design in Digital Health.

Using the results presented in this paper, several recommendations can be extracted to help digital health design teams maximize the value of patient-public co-design in digital health design. These recommendations have been summarized below and in Fig 2.

The first set of recommendations relate to how design teams should plan and prepare for patient-public co-design. These recommendations include (1) prioritizing equity-based co-design recruitment and planning (i.e., recruiting co-design partners who are representative of the target population, and ensuring engagement methods are adapted to the needs of these partners) and (2) reconsidering assumptions about co-design process needs (i.e., working collaboratively with partners to re-define co-design goals, identify solutions partners already utilize, and co-create a communication plan). Because the co-design process appears to be empowering intervention itself, it is recommended that design teams put considerable time and effort into planning and preparing appropriate and equitable strategies. Substantial research has been done to amalgamate best-practices for facilitating equity in digital health co-design, including work from Chauhan et al.[37], Moll et al.[38], and Brewer et al.[39]. For instance, Chauhan et al. [37] recommends ensuring seldom-heard populations are invited by liaising with local communities and support groups, while considering peer-led engagement strategies. Chauhan et al. [37] also recommend allocating adequate resources for co-design, including financial renumeration and accessibility considerations. Chauhan et al. recommend developing a co-design term of reference and a co-design evaluation plan. Moll et al. [38] build on Chauhan et al. [37] by proposing reflexivity questions that design teams can ask themselves. The first grouping of questions pushes design teams to reflect on where they are starting from, including their worldviews, beliefs, power, and privilege. The second grouping of questions pushes design teams to reflect on what they will be doing during co-design, including how they will achieve diverse representation, what tools will be used to understand people’s lived experiences, and how they will uncover the knowledge from diverse perspectives. The final grouping of questions pushes design teams to reflect on what the intended outputs should be, including how to plan for implementation, how to build capacity, and how to measure success. Brewer et al. [39] suggest best-practices for equitable design of digital health interventions across eight domains. These domains include recruitment and retention of diverse populations, leveraging established stakeholders and trusted social networks, understanding the social context of potential end users and populations, integrating community engagement through user-centered design, gaining an understanding of community partner technology infrastructure, planning time and resources to devote to community engagement processes. Overall, the results of this paper corroborate the recommendations of Chauhan et al., Moll et al., and Brewer et al., and advance their work by suggesting that the planning and preparation stage of co-design is essential in order to maximize the value of patient-public contributions.

The second set of recommendations relate to how design teams should approach the design process of patient-public co-design. These recommendations include (1) engaging co-design partners in *product* design (i.e., utilizing co-design partner insights to design a digital health product with an engaging user experience, relevant behaviour change supports, and appropriate in-person intervention integration) and (2) engaging co-design partners in *implementation* design (i.e., utilizing co-design partner insights to plan for digital health product implementation, including how users will perceive a need for the solution, seek the solution, reach the solution, and adopt the solution). The results of this paper suggest that to maximize the value of co-design, design teams should be much more considerate of the multiple ways patient-public partners can be involved in design. In a 2016 review of co-design approaches for digital health, Eyles et al. mapped out the methods (e.g., focus groups, surveys, storyboarding, observations, and workshops) and phases (e.g., background knowledge, user needs, intervention content, prototyping, and piloting) of digital health co-design [8]. Although this review provided practical information for digital health design teams, our paper advances this review by specifying exactly what should be co-designed, spanning product and implementation design. Regarding product design, Cole Lewi et al. argue that designing effective digital health products necessitates designing (a) an engaging user interface and user experience and (b) evidence-based behaviour change techniques [40]. Yardley et al. share similar recommendations and highlight the importance of designing human support strategies and tailoring capabilities [41]. Although this paper corroborates the importance of co-designing engaging user interfaces, relevant behaviour change supports, and appropriate in-person integration, it also advances Yardley et al. and Cole-Lewis et al.’s work. Our paper recommends moving beyond co-designing effective *products*, and toward co-designing technology-supported *services*. Our paper highlights the importance of systematically embedding implementation planning into the digital health behaviour change design process; an idea advocated in the “service design” literature [42,43]. A recent paper by Shaw et al. argues that the inter-relation between product innovation and service innovation for digital health has not been sufficiently acknowledged, which is contributing to ongoing challenges of technology adoption [43]. Aligning with Shaw et al.’s comments, our paper suggests that there needs to be a back-and-forth between co-designing an effective digital health product (i.e., digital feature and content design) and an effective digital health service (i.e., innovations in teams, processes, and routines).

The last set of recommendations relate to how design teams should utilize co-design to ensure the ongoing adaptation and sustainability of digital health innovations. These recommendations include (1) co-designing equity-informed data collection and tailoring capabilities (i.e., utilizing co-design partner insights to identify equity-informed data collection measures that can be used to create an adaptable solution that will meet users’ needs over time) and (2) co-designing how the intervention will be sustained over time (i.e., working collaboratively with co-design partners to decide on how the digital health intervention can be championed and maintained in an evolving health system). The results of this paper suggest that maximizing the value of co-design necessitates ensuring that the co-design process does not end once the digital health intervention has been designed. Co-design should continue throughout the lifecycle of the product to ensure that the product is adapted to meet users’ changing needs and sustained within health systems in flux. A recent review by van Kessel et al. mapped the factors affecting the widespread uptake of digital health innovations in health systems [44]. Their review suggests that some of the frequently reported factors affecting uptake include developing sustainable funding options, robust digital infrastructure, novel professional guidelines and protocols, ongoing digital health training, interoperability across the health system, and tailoring to account for different needs across populations and disease stages [44]. The results of our paper corroborate the findings of van Kessel et al., and suggest that the value of co-design would be diminished if patient-public insights are not used to foster the viability of digital health innovation.

### Limitations and future directions

Although our research team attempted to recruit a diverse range of interviewees based on interviewee type, location, and digital health design experience, the biggest limitation of this study related to the diversity of the sample. Our interviewee sample was unequally balanced with 19 design leaders and 9 patient-public partners. Although this imbalance was not ideal, we believe the richness in the data collected from our patient-public partners was comparable to that of our design leaders, allowing us to meaningfully address our research objective with data from both interviewee groups. In addition to this imbalance, our sample only consisted of participants from North America, Europe, and Oceania. The lack of participants from low-income, equity-deserving populations was notable. Our omission of collecting pre-interview demographic data (e.g., on race or gender) limited our ability to better understand the representativeness of our sample. Many digital health solutions fail to design for the challenges faced by equity-deserving groups, especially racial and ethnic minorities [39]. Concern has been raised in the patient-public engagement literature about issues of representativeness in patient-public partners [36]. Future research may benefit from taking an equity, diversity, and inclusion lens to explore the value of patient and public involvement in digital health design. It should also be noted that because our recruitment of design leaders focused on those who had designed digital health interventions for patient-public health *behaviour change*, many design leaders had background in behavioral science. Although we feel that a design leader sample with expertise in human behavior added richness to our findings, future research may benefit from studying how design leaders from different academic backgrounds perceive the value of patients and public involvement.

### Conclusions

This paper aimed to clarify how the value of patient and public involvement in digital health design can be maximized, from the perspective of digital health design leaders and patient-public partners. Digital health design teams may benefit from using the findings in this paper to prepare for how they will involve patients and the public in collaborative design in the future. Future research may benefit from clarifying the barriers and facilitators to ensuring these value-adders are realized.

## Supporting information

S1 Appendix “Participant Information”. (DOCX)
