# Supplementary material for: Maximizing the value of patient and public involvement in the digital health co-design process: A qualitative descriptive study with design leaders and patient-public partners
Source: PLOS Digit Health. 2023 Oct 25;2(10):e0000213. doi: 10.1371/journal.pdig.0000213 (PMC10599516; doi:10.1371/journal.pdig.0000213)
Supplement: S1 Appendix — (DOCX) [file pdig.0000213.s001.docx]

**S1 Appendix: Participant Information**

| **Design Leader Interviewees** | | | |
| --- | --- | --- | --- |
| **Name** | **Location** | **Type of Digital Health Project** | **Job Title** |
| Aline Holzwarth | USA | Multiple different types | Behavioral Science Lead, Apple, Health AI |
| Amelia Hyatt | Australia | Cancer Consultation Recording Application | Health Researcher, Peter MacCallum Cancer Centre |
| Amy Bucher | USA | Multiple different types | Chief Behavioural Officer, Lirio |
| Bart Pouls | Netherlands | Rheumatoid Arthritis Drug Adherence Application | Health Researcher and Pharmacist, Sint Maartenskliniek |
| Christian Jensen | Denmark | Musculoskeletal Pain Management Application | Software Engineer, Trade eXpansion |
| Dustin DiTommaso | USA | Multiple different types | Chief Design Office, meQuilibrium |
| Guido Giunti | Finland | Multiple different types | Physician and Digital Health Leader, University of Oulu |
| Holly Witteman | Canada | Multiple different types | Canada Research Chair in Human-Centred Digital Health |
| Luis Fernandez-Luque | Spain | Adaptive self-management for people living with chronic conditions | Chief Scientific Officer at Adhera Health |
| Lydia Sequeira | Canada | Mental Health and Suicide Prevention Applications | Health Researcher, Centre for Addictions and Mental Health and Canada Health Infoway |
| Malene Jagd Svendsen | Denmark | Musculoskeletal Pain Management Application | Health Researcher, University of Southern Denmark |
| Pauline Kabitsis | Canada | Multiple different types | Applied Behavioural Scientist, Common Thread |
| Robyn Whittaker | New Zealand | Multiple different types | Physician and Digital Health Researcher, National Institute for Health Innovation |
| Ruth Schmidt | USA | Multiple different types | Associate Professor at the Institute of Design (ID) |
| Samuel Salzer | Sweden | Multiple different types | Behavioral Science Advisor |
| Soren Kleberb | Denmark | Musculoskeletal Pain Management Application | CEO of SelfBack |
| Anonymous | USA | Physical Activity Application for General Wellbeing | Health Researcher at a University |
| Anonymous | Netherlands | Physical Activity Application for General Wellbeing | Health Researcher at a University |
| Anonymous | USA | Multiple different types | Director at Health Technology Research Centre |

| **Patient/Public Interviewees** | | |
| --- | --- | --- |
| **Name** | **Location** | **Type of Digital Health Project** |
| Agni Shah | Canada | Hospital to Home Transition Application |
| Alan White | Australia | Cancer Applications |
| Barb Sklar | Canada | Multimorbidity Self-Management Application |
| Dana Arafeh | Canada | Multiple |
| Fiona White | Australia | Cancer Applications |
| Jane Liu | Canada | Hospital to Home Transition Application |
| Mary Hynes | Canada | Multimorbidity Self-Management Application |
| Anonymous | Canada | Cancer Rehabilitation Application |
| Anonymous | Canada | Cancer Rehabilitation Application |
